# Comparative Analysis of Gradient Diffusion and Disk Diffusion with Agar Dilution for Susceptibility Testing of *Elizabethkingia anophelis*

**DOI:** 10.3390/antibiotics10040450

**Published:** 2021-04-16

**Authors:** Chien-Tung Chiu, Chung-Hsu Lai, Yi-Han Huang, Chih-Hui Yang, Jiun-Nong Lin

**Affiliations:** 1 Department of Critical Care Medicine, E-Da Hospital, I-Shou University, Kaohsiung 824, Taiwan; chiuct168@gmail.com; 2 Department of Internal Medicine, Division of Infectious Diseases, E-Da Hospital, I-Shou University, Kaohsiung 824, Taiwan; laich6363@yahoo.com.tw; 3 School of Medicine, College of Medicine, I-Shou University, Kaohsiung 824, Taiwan; je091410show@hotmail.com; 4 Department of Biological Science and Technology, Meiho University, Pingtung 912, Taiwan; puppylovefu@gmail.com

**Keywords:** *Elizabethkingia anophelis*, antimicrobial susceptibility testing, agar dilution, gradient diffusion, disk diffusion

## Abstract

*Elizabethkingia anophelis* has recently emerged as a cause of life-threatening infections. This study compared the results of antimicrobial susceptibility testing (AST) conducted for *E. anophelis* through different methods. *E. anophelis* isolates collected between January 2005 and June 2019 were examined for their susceptibility to 14 antimicrobial agents by using disk diffusion, gradient diffusion (Etest; bioMérieux S.A., Marcy l’Etoile, France), and agar dilution methods. The agar dilution method was the reference assay. According to the agar dilution method, the isolates exhibited the highest susceptibility to minocycline (100%), doxycycline (97.6%), rifampin (95.2%), and levofloxacin (78.6%). A very major error rate of >1.5% was observed for nine antibiotics tested using the disk diffusion method. The overall categorical agreement rate between the disk diffusion and agar dilution methods was 74.8%, and ceftazidime, minocycline, levofloxacin, and rifampin met the minimum requirements for discrepancy and agreement rates. The Etest method tended to produce lower log_2_ minimum inhibitory concentrations for the antibiotics, except for trimethoprim–sulfamethoxazole and rifampin; the method resulted in very major errors for nine antibiotics. The overall essential and categorical agreement rates between the Etest and agar dilution methods were 67.3% and 76.1%, respectively. The Etest method demonstrated acceptable discrepancy and agreement rates for ceftazidime, minocycline, doxycycline, levofloxacin, and rifampin. AST results obtained through the disk diffusion and Etest methods for multiple antibiotics differed significantly from those obtained using the agar dilution method. These two assays should not be a routine alternative for AST for *E. anophelis*.

## 1. Introduction

Members of the genus *Elizabethkingia* are aerobic, Gram-negative, nonfermenting, nonmotile, catalase-positive, oxidase-positive, and indole-positive bacilli distributed in natural soil and water environments [1,2,3,4]. Since its first identification by Elizabeth O. King in 1959 [4], *Elizabethkingia* has been reported to cause human infections. Currently, six species constitute the genus *Elizabethkingia*, namely, *E. meningoseptica*, *E. miricola*, *E. anophelis*, *E. bruuniana*, *E. ursingii*, and *E. occulta* [5]. *E. anophelis* was initially isolated in the midgut of the *Anopheles gambiae* mosquito in 2011 [6], and this pathogen has been identified to be the species most commonly associated with human infections, particularly in immunocompromised patients [7,8,9,10,11,12,13]. The overall mortality rate of patients infected with *E. anophelis* ranges from 24% to 60% [7,8,9,10,11,12,13]. Several outbreaks of life-threatening infections caused by *E. anophelis* have been described in many countries, including Singapore, Hong Kong, South Korea, Taiwan, and the United States [7,8,9,10,11,12,13].

Several studies have reported that *E. anophelis* strains typically expressed resistance to multiple antibiotics, but others have revealed that they demonstrated susceptibility to some antibiotics such as certain β-lactams, fluoroquinolones, and sulfa drugs [7,8,9,10,11,12,13]. These inconsistent antimicrobial susceptibility testing (AST) results can be attributed to the use of different testing methods, such as disk diffusion, gradient diffusion, broth dilution, and agar dilution assays.

According to Clinical and Laboratory Standards Institute (CLSI) guidelines [14], breakpoints of antimicrobial agents against “other non-Enterobacteriaceae” are only determined using broth dilution or agar dilution minimum inhibitory concentration (MIC) testing. Despite the inconsistent AST results reported by numerous studies, no study has evaluated the accuracy of non-reference testing methods for *E. anophelis*. To fill this research gap, we used the CLSI-recommended standard agar dilution method as a reference assay and examined the concordance of AST results obtained from gradient diffusion and disk diffusion methods for clinical *E. anophelis* isolates.

## 2. Materials and Methods

### 2.1. Ethics

This study was approved by the Institutional Review Board of E-Da Hospital, an approximately 1000-bed university-affiliated medical center in Kaohsiung, Taiwan (EMRP-107-139). All experiments in this study were conducted in accordance with the Declaration of Helsinki and national standards of Taiwan. The need for patient informed consent was waived because the AST of microorganisms that were obtained from routine clinical cultures involved minimal risk of harm to patients.

### 2.2. Study Setting and Design

The database of clinical microbiology laboratory of E-Da Hospital was searched for microbial cultures that yielded *Elizabethkingia* species from January 2005 to June 2019. All isolates used in this study were routinely collected from patients according to their clinical requirements. These isolates were reserved as glycerol stocks at −80 °C until use. Accurate species of the stored *Elizabethkingia* isolates were re-identified through 16S ribosomal ribonucleic acid gene sequencing as described in our previous study [11]. Isolates recognized as *E. anophelis* were included in this study. The MICs and antimicrobial susceptibility patterns of 14 antimicrobial agents against all *E. anophelis* isolates were concomitantly determined using the agar dilution, gradient diffusion, and disk diffusion methods. The CLSI-recommended agar dilution method was used as a reference assay.

### 2.3. Agar Dilution Assay

*MICs* obtained using the agar dilution method were performed in accordance with CLSI guidelines [15]. In brief, the isolates were spread on Mueller–Hinton agar plates (Becton Dickinson, Sparks, MD, USA) for overnight culture at 35 °C; subsequently, new colonies were inoculated into Mueller–Hinton broth tubes. Bacterial suspensions were adjusted to a turbidity equivalent to that of a 0.5 McFarland standard. AST plates (9 cm in diameter) were prepared by adding 14 serially twofold-diluted antibiotics to Mueller–Hinton agar plates (Table 1). A bacterial suspension of 1 µL (approximately 10^5^ colony-forming unit (CFU)/mL) from a prepared 0.5 McFarland standard suspension was added as a spot, and 2 spots per strain were added to each plate. Twenty-four strains were tested per plate. These plates were incubated at 35 °C in ambient air for 16–20 h.

### 2.4. Disk Diffusion Assay

Disk susceptibility tests were performed using the CLSI-recommended standard technique [16]. The suspensions 0.5 McFarland standard were swabbed onto the Mueller–Hinton agar plates. Filter paper disks (Becton Dickinson) impregnated with the 14 antimicrobial agents tested in this study were placed on top of the agar plates; Table 1 lists the concentrations of antimicrobial agents impregnated on the disks. These plates were incubated at 35°C in ambient air for 16–20 h. After incubation, the size of the inhibition zone around each disk was measured.

### 2.5. Gradient Diffusion Assay

The gradient diffusion assay was performed using Etest according to the manufacturer’s instructions (bioMérieux S.A., Marcy l’Etoile, France). The bacterial suspension preparation, inoculum, and incubation procedures were the same as those used for the disk diffusion method. Table 1 presents the gradient concentrations of the 14 antibiotics on the strips.

### 2.6. Susceptibility Interpretation

The MIC interpretive criteria for the results of agar dilution and Etest methods were adopted from CLSI standards for “other non-Enterobacteriaceae” [14], except for tigecycline, rifampin, and vancomycin. The disk diffusion breakpoints were not established for “other non-Enterobacteriaceae” by CLSI. The susceptibility results by the disk diffusion method were determined according to the CLSI diameter interpretive criteria for Acinetobacter species because their MIC breakpoints were the same as those of “other non-Enterobacteriaceae” [14]. The susceptibility testing results of tigecycline were interpreted according to Enterobacteriaceae breakpoints provided by the U.S. Food and Drug Administration (FDA) (MIC: susceptible, ≤2 mg/L; intermediate, 4 mg/L; resistant, ≥8 mg/L; zone diameter: susceptible, ≥19 mm; intermediate, 15–18 mm; resistant, ≤14 mm) [17]. For rifampin and vancomycin, the susceptibility criteria of zone diameters and MICs were interpreted according to CLSI standards for Enterococcus species [14].

### 2.7. Comparative Performance Data

The agreement and discrepancy among the different testing methods were evaluated as described previously [18,19]. The very major error (discrepancy) rate was defined as the percentage of the number of isolates with false-susceptible results divided by the number of resistant isolates; major error (discrepancy) rate was defined as the percentage of the number of isolates with false-resistant results divided by the number of susceptible isolates, and minor error (discrepancy) rate was defined as the percentage of the number of isolates with intermediate results from the evaluated method and susceptible or resistant results from the reference method, or vice versa, divided by the total number of isolates. The essential agreement rate was defined as the percentage of the number of isolates with MICs—provided by the evaluated method—within ±1 log_2_ dilution of the reference MIC divided by the number of isolates tested. The categorical agreement rate was defined as the percentage of the number of isolates—tested using the evaluated method—with the same categorical interpretation (susceptible, intermediate, and resistant) as the reference divided by the total number of isolates examined. According to U.S. FDA criteria [18,19], the acceptable values of the very major error, major error, and minor error rates are ≤1.5%, ≤3%, and ≤10%, respectively. Both essential and categorical agreement rates are considered acceptable if they are ≥90%.

## 3. Results

### 3.1. Susceptibility

According to the CLSI-recommended agar dilution method, the susceptibility rates were as follows: minocycline, 100%; doxycycline, 97.6%; rifampin, 95.2%; levofloxacin, 78.6%; ciprofloxacin, 8.3%; tigecycline, 4.8%; trimethoprim–sulfamethoxazole, 1.2%; and others, 0%. The disk diffusion method demonstrated that the among the 14 antibiotics, the isolates exhibited adequate susceptibility to minocycline (100%), doxycycline (98.8%), levofloxacin (79.8%), and rifampin (85.7%; Table 2). As revealed by the Etest method, the isolates exhibited high rates of susceptibility to minocycline (100%), doxycycline (97.6%), tigecycline (77.4%), ciprofloxacin (75%), levofloxacin (79.8%), and rifampin (94%) but showed extremely low rates of susceptibility to the other antibiotics (<15%).

### 3.2. MIC Determination

A total of 84 *E. anophelis* isolates were included for AST in which the disk diffusion, Etest, and agar dilution methods were concomitantly performed. Table 2 presents the antimicrobial susceptibility patterns. In general, the Etest method tended to yield lower MICs than did the agar dilution method for most antimicrobial agents. For example, the MIC of tigecycline determined using the Etest method ranged from 0.125 to 8 mg/L, whereas that determined using the agar dilution method ranged from 2 to 32 mg/L. The MIC_50_ levels for the following antibiotics were obtained using the Etest and agar dilution methods, respectively: minocycline, 0.125 and 0.25 mg/L; doxycycline, 1 and 2 mg/L; tigecycline, 1.5 and 8 mg/L; ciprofloxacin, 0.5 and 2 mg/L; levofloxacin, 0.38 and 2 mg/L; and rifampin, 0.38 and 0.5 mg/L. Of the 14 antibiotics, minocycline was the most active against *E. anophelis* and had the lowest MIC_50_ and MIC_90_ determined using both the Etest and agar dilution methods.

### 3.3. Discrepancy and Agreement Rates between Disk Diffusion and Agar Dilution Methods

**Figure 1:**
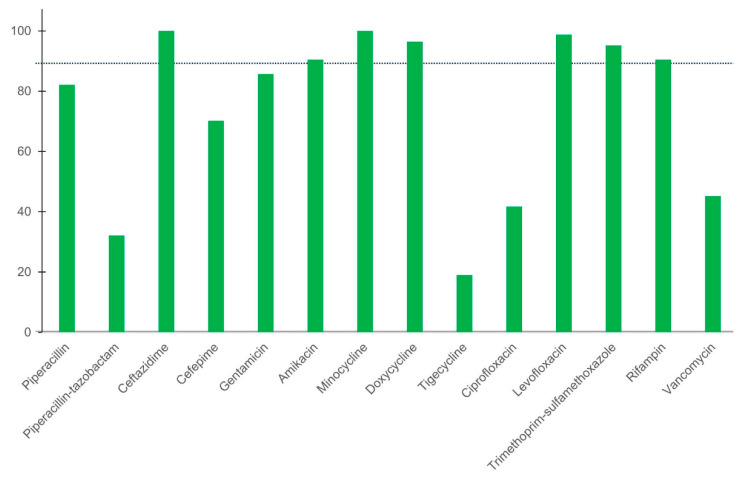
Categorical agreement between the disk diffusion and agar dilution methods for the antimicrobial susceptibility testing of *E. anophelis*. The broken line indicates an agreement rate of 90%.

The results revealed a very major error rate of <1.5% for ceftazidime, minocycline, levofloxacin, and rifampin; the very major error rate was also noted to be high for the other antibiotics (Table 3). The disk diffusion method indicated particularly high, very major error rates for piperacillin–tazobactam (22.6%), cefepime (21.4%), doxycycline (100%), tigecycline (50.9%), and vancomycin (25.3%). Trimethoprim–sulfamethoxazole was the only antibiotic with a major error (100%). The results also revealed acceptable categorical agreement rates (>90%) for ceftazidime, amikacin, minocycline, doxycycline, levofloxacin, trimethoprim–sulfamethoxazole, and rifampin (Figure 1). The overall rate of categorical agreement between the disk diffusion and agar dilution methods was 74.8%. Of the 14 antimicrobial agents, only ceftazidime, minocycline, levofloxacin, and rifampin met the minimum requirements for discrepancy and categorical agreement rates.

### 3.4. Discrepancy and Agreement Rates between Etest and Agar Dilution Methods

**Figure 2:**
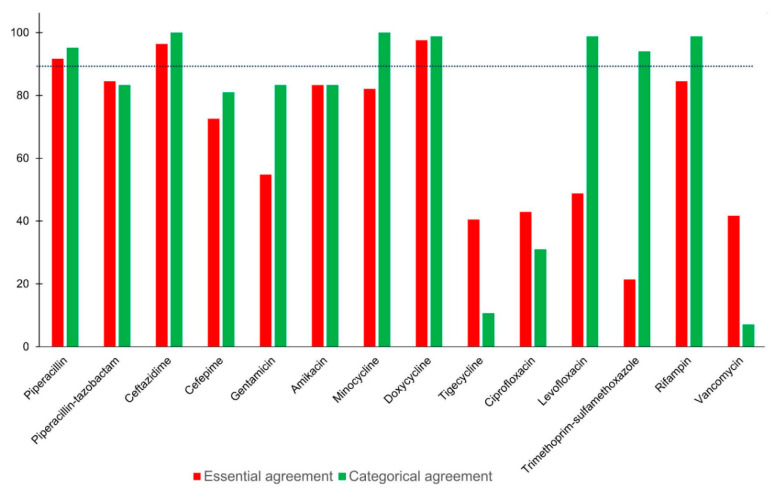
Essential agreement and categorical agreement between the Etest and agar dilution methods for the antimicrobial susceptibility testing of *E. anophelis*. The broken line indicates an agreement rate of 90%.

Compared with the agar dilution method, the Etest method tended to produce lower log_2_ MICs for the antimicrobial agents, except for trimethoprim–sulfamethoxazole and rifampin (Table 3). The essential agreement rates were acceptable (≥90%) only for piperacillin, ceftazidime, and doxycycline (Figure 2). By contrast, the essential agreement rates were substantially low for tigecycline, ciprofloxacin, levofloxacin, trimethoprim–sulfamethoxazole, and vancomycin (<50%). Acceptable very major error rates (<1.5%) were determined for ceftazidime, minocycline, doxycycline, levofloxacin, and rifampin. Similar to the results obtained using the disk diffusion method, trimethoprim–sulfamethoxazole was the only agent with a major error (100%). The rate of categorical agreement (>90%) between the two methods was high for piperacillin (95.2%), ceftazidime (100%), minocycline (100%), doxycycline (98.8%), levofloxacin (98.8%), trimethoprim–sulfamethoxazole (94%), and rifampin (98.8%) (Figure 2). The categorical agreement rate was extremely low for tigecycline (10.7%), ciprofloxacin (31%), and vancomycin (7.1%). The overall rates of essential and categorical agreement between the Etest and agar dilution methods were 67.3% and 76.1%, respectively. Ceftazidime, minocycline, doxycycline, levofloxacin, and rifampin demonstrated acceptable discrepancy and agreement rates.

## 4. Discussion

The antimicrobial susceptibility of *E. anophelis* has been reported by studies conducted in several countries, including Singapore [7,8], Hong Kong [9], South Korea [10], Taiwan [11,12], and the United States [13]. These studies have concurrently demonstrated *E. anophelis* to exhibit drug resistance to most β-lactams, β-lactam/lactamase inhibitor combinations, aminoglycosides, and carbapenems, irrespective of the testing techniques used. However, inconsistent AST results were reported for some antibiotics. According to the results of the CLSI-recommended agar dilution assay, we examined the multidrug-resistant characteristics of *E. anophelis*. Among the 14 antimicrobial agents tested in this study, only minocycline, doxycycline, rifampin, and levofloxacin were mostly effective against *E. anophelis*. These 14 antibiotics tested exhibit their bacteriostatic or bactericidal activity via different mechanisms. β-Lactam antibiotics (piperacillin, piperacillin–tazobactam, ceftazidime, and cefepime) target the penicillin-binding proteins and kill bacteria by inhibiting the synthesis of cell walls. Aminoglycosides (gentamicin and amikacin) and tetracyclines/glycylcycline (minocycline, doxycycline, and tigecycline) inhibit protein synthesis by binding to the 30S ribosomal subunit. Fluoroquinolones (ciprofloxacin and levofloxacin) act by inhibiting DNA topoisomerases (DNA gyrase and topoisomerase IV). Sulfamethoxazole inhibits the synthesis of dihydrofolic acid and trimethoprim inhibits thymidine and DNA synthesis. Rifampicin inhibits bacterial DNA-dependent RNA polymerase. Vancomycin binds to the D-Ala-D-Ala of cell walls and inhibits cell wall synthesis [20]. With regard to rifampin, it is known that rifampin is less active against Gram-negative bacilli because it does not readily penetrate the outer membrane of these bacteria [21]. The mechanism for the low rifampin MICs of *E. anophelis* is not clear and warrants further investigation.

The disk diffusion method is an inexpensive, convenient, and excellent AST approach for many microorganisms. Nevertheless, our study revealed that this method exhibited poor performance in the AST of *E. anophelis*. Only ceftazidime, minocycline, levofloxacin, and rifampin were considered acceptable for AST executed through the disk diffusion method. Moreover, the most critical concern of using the disk diffusion method for *E. anophelis* AST is the unacceptable high rate of very major errors.

In the Etest method, the antibiotic gradient diffusion strips constitute a convenient tool for AST. Our study revealed acceptable rates of error and agreement between the Etest and agar dilution methods for ceftazidime, minocycline, doxycycline, levofloxacin, and rifampin. Among these antibiotics, only minocycline, doxycycline, levofloxacin, and rifampin were potentially effective against *E. anophelis*. Moreover, we observed a considerable discrepancy and a poor rate of agreement between the Etest and agar dilution methods. The Etest method underestimated the MICs of all antimicrobial agents tested in this study, except for rifampin. The tendency of the Etest to underestimate MICs has been reported for other microorganisms [22,23]. This false-susceptible predisposition resulted in high rates of very major errors for many antibiotics tested. This is a critical concern for clinicians because patients might be inappropriately treated and die due to these false-susceptible testing results.

When investigating 25 *E. anophelis* isolates from the Wisconsin outbreak, Perrin et al. [13] used the disk diffusion method for AST determination and found susceptibility to piperacillin (100%), piperacillin–tazobactam (92%), cefepime (92%), and ciprofloxacin (92%). In addition, Lau et al. [9] examined 17 *E. anophelis* isolates from Hong Kong by using the disk diffusion method and demonstrated the following susceptibility rates for various agents: ceftazidime, 100%; ciprofloxacin, 100%; trimethoprim–sulfamethoxazole, 70.6%; and vancomycin (100%). These AST results obtained using the disk diffusion method are nearly contradictory to our findings, which indicate that *E. anophelis* isolates were completely resistant to these antibiotics. Moreover, vancomycin cannot penetrate the out membrane to access the D-Ala-D-Ala binding sites on cell wall, and therefore it is not effective against Gram-negative bacteria, except some *Neisseria* species [24]. Our study revealed a high very major error rate and low categorical agreement rate for these antibiotics through the disk diffusion method. These differences in susceptibility between the studies could be attributed to the geographic variation as well as false susceptibility.

## 5. Conclusions

*E. anophelis* has recently become an emerging life-threatening infection in humans. Accurately evaluating antimicrobial susceptibility is imperative for patient care. We suggest that the disk diffusion and Etest methods are not an acceptable alternative for all AST approaches for *E. anophelis*. AST through the disk diffusion and Etest methods could be reliably performed for only some antibiotics.

## Figures and Tables

**Table 1:** The ranges of antimicrobial concentrations used in this study.

Antimicrobial Agent ^a^	Susceptibility Testing Assay		
	Agar Dilution (mg/L)	Disk Diffusion (µg)	Etest (mg/L)
Piperacillin	2–256	100	0.016–256
Piperacillin–tazobactam	2/4–256/4	100/10	0.016/4–256/4
Ceftazidime	8–256	30	0.016–256
Cefepime	1–128	30	0.016–256
Gentamicin	1–128	10	0.016–256
Amikacin	2–256	30	0.016–256
Minocycline	0.0625–2	30	0.016–256
Doxycycline	0.25–16	30	0.016–256
Tigecycline	0.25–32	15	0.016–256
Ciprofloxacin	0.25–32	5	0.002–32
Levofloxacin	0.25–32	5	0.002–32
Trimethoprim–sulfamethoxazole	0.5/9.5–32/608	1.25/23.75	0.002/0.38 – 32/608
Rifampin	0.25–16	5	0.016–256
Vancomycin	2–256	30	0.016–256

^a^ Sources of standard powders: ciprofloxacin and levofloxacin were obtained from Sigma-Aldrich (St. Louis, MO, USA), and others were bought from Cyrusbioscience (Taipei, Taiwan).

**Table 2:** Antimicrobial MICs (mg/L) and susceptible rates of 84 *E. anophelis* isolates determined using the agar dilution, Etest, and disk diffusion methods.

Antimicrobial Agent	Agar Dilution				Disk Diffusion	Etest			
	MIC Range	MIC_50_	MIC_90_	% S^a^	% S^a^	MIC Range	MIC_50_	MIC_90_	% S^a^
Piperacillin	128–>256	>256	>256	0	4.8	8–>256	>256	>256	2.4
Piperacillin–tazobactam	256/4–>256/4	>256/4	>256/4	0	22.6	8/4–>256/4	>256/4	>256/4	4.8
Ceftazidime	256–>256	>256	>256	0	0	48–>256	>256	>256	0
Cefepime	32–>128	>128	>128	0	21.4	1.5–>256	>256	>256	2.4
Gentamicin	8–>128	>128	>128	0	9.5	3–>256	64	>256	13.1
Amikacin	32–>256	>256	>256	0	6	12–>256	>256	>256	3.6
Minocycline	0.125–1	0.25	0.25	100	100	0.023–0.38	0.125	0.19	100
Doxycycline	1–16	2	4	97.6	98.8	0.5–16	1	2	97.6
Tigecycline	2–32	8	16	4.8	56	0.125–8	1.5	3	77.4
Ciprofloxacin	1–>32	2	>32	8.3	51.2	0.038–>32	0.5	>32	75
Levofloxacin	0.5–>32	2	32	78.6	79.8	0.038–>32	0.38	>32	79.8
Trimethoprim–sulfamethoxazole	2/38–32/608	8/152	16/304	1.2	1.2	0.38/7.22–>32/608	>32/608	>32/608	4.8
Rifampin	<0.25–>16	0.5	1	95.2	85.7	0.125–>32	0.38	0.75	94
Vancomycin	8–256	32	64	0	25	3 –3 2	8	16	9.5

^a^ S, susceptible.

**Table 3:** Comparison of the disk diffusion and Etest methods with the agar dilution method for antimicrobial susceptibility testing of 84 *E. anophelis* isolates.

Antimicrobial Agent	Technique	Isolate no. of MIC log_2_ Dilutions Differ from the Agar Dilution							Essential Agreement (%)	Interpretive Errors (%)			Categorical Agreement (%)
		≤−3	−2	−1	0	+1	+2	≥+3		Very Major	Major	Minor	
Piperacillin	Disk diffusion	–	–	–	–	–	–	–	–	4.8	N/A	13.1	82.1
	Etest	4	1	1	55	21	2	0	91.7	2.4	N/A	2.4	95.2
Piperacillin–tazobactam	Disk diffusion	–	–	–	–	–	–	–	–	22.6	N/A	45.2	32.1
	Etest	7	5	3	48	20	1	0	84.5	4.8	N/A	11.9	83.3
Ceftazidime	Disk diffusion	–	–	–	–	–	–	–	–	0	N/A	0	100
	Etest	3	0	1	77	3	0	0	96.4	0	N/A	0	100
Cefepime	Disk diffusion	–	–	–	–	–	–	–	–	21.4	N/A	8.3	70.2
	Etest	19	4	3	58	0	0	0	72.6	2.4	N/A	16.7	81
Gentamicin	Disk diffusion	–	–	–	–	–	–	–	–	9.6	N/A	4.8	85.7
	Etest	17	21	19	25	2	0	0	54.8	13.3	N/A	3.6	83.3
Amikacin	Disk diffusion	–	–	–	–	–	–	–	–	4.8	N/A	4.8	90.5
	Etest	3	8	9	45	16	3	0	83.3	3.6	N/A	13.1	83.3
Minocycline	Disk diffusion	–	–	–	–	–	–	–	–	0	0	0	100
	Etest	2	13	42	24	3	0	0	82.1	0	0	0	100
Doxycycline	Disk diffusion	–	–	–	–	–	–	–	–	100	0	1.2	96.4
	Etest	0	2	32	43	7	0	0	97.6	0	0	1.2	98.8
Tigecycline	Disk diffusion	–	–	–	–	–	–	–	–	50.9	0	48	19
	Etest	25	25	29	5	0	0	0	40.5	71.7	0	44	10.7
Ciprofloxacin	Disk diffusion	–	–	–	–	–	–	–	–	15.2	0	52.4	41.7
	Etest	18	30	16	19	1	0	0	42.9	39.4	0	53.6	31
Levofloxacin	Disk diffusion	–	–	–	–	–	–	–	–	0	0	1.2	98.8
	Etest	13	20	26	9	6	0	0	48.8	0	0	1.2	98.8
Trimethoprim–sulfamethoxazole	Disk diffusion	–	–	–	–	–	–	–	–	1.2	100	2.4	95.2
	Etest	3	2	6	8	4	22	39	21.4	4.8	100	0	94
Rifampin	Disk diffusion	–	–	–	–	–	–	–	–	0	0	9.5	90.5
	Etest	0	0	14	38	19	13	0	84.5	0	0	1.2	98.8
Vancomycin	Disk diffusion	–	–	–	–	–	–	–	–	25.3	NA	32.1	45.2
	Etest	8	41	33	2	0	0	0	41.7	5.3	NA	88.1	7.1
